# A National Database Analysis: Does Cold Weather Affect the Surgical Intervention Rate for Developmental Dysplasia of the Hip in Children Under Five Years?

**DOI:** 10.7759/cureus.57998

**Published:** 2024-04-10

**Authors:** Samantha L Ferraro, Anthony K Chiu, B. Tanner Seibold, Alex Gu, Amil R Agarwal, Sarah Dance, Savyasachi C Thakkar, Sean Tabaie

**Affiliations:** 1 Orthopaedic Surgery, George Washington University School of Medicine and Health Sciences, Washington DC, USA; 2 Orthopaedic Surgery, Children’s National Hospital, Washington DC, USA; 3 Orthopaedic Surgery, Johns Hopkins Health System, Baltimore, USA; 4 Orthopaedic Surgery, Children's National Hospital, Washington DC, USA

**Keywords:** cold weather, open hip reduction, adductor tenotomy, osteotomy, pediatric hip, developmental dysplasia of the hip

## Abstract

Background

Cold weather in the first few months of life may increase the risk of a late diagnosis of developmental dysplasia of the hip (DDH). Early detection of DDH can often be treated non-surgically. The purpose of this study is to observe whether the rates of surgical intervention for DDH differ based on average outdoor temperatures in the winter months.

Methods

A retrospective observational study of DDH patients diagnosed from 2010 to 2021 was conducted using a national administrative database. Five geographic regions were defined based on the average temperatures in the coldest quarter of the year. The rates of DDH-related surgeries were compared across these temperature regions.

Results

A total of 55,911 patients ≤5 years old with a DDH diagnosis from 2010 to 2021 were identified in the database. When compared to the warmest region (Group 5), the coldest region (Group 1) had higher rates of open reduction (4.59% vs. 2.06%, p<0.001), adductor tenotomy (6.95% vs. 2.91%, p<0.001), femoral osteotomy (5.75% vs. 2.04%, p<0.001), pelvic osteotomy (5.27% vs. 2.04%, p<0.001), and total DDH surgeries (11.42% vs. 5.03%, p<0.001).

Conclusion

Children living in states with an average winter temperature of -6.17°C had an increased likelihood of requiring surgical intervention for DDH within the first five years of life.

## Introduction

Developmental dysplasia of the hip (DDH) is the most common congenital condition in newborns [1], affecting at least one in 1,000 live births [2-4]. When DDH is diagnosed early, generally considered to be at age six months or younger [3-5], the utilization of a Pavlik harness or hip spica casting has a high success rate. However, if diagnosed later, successful treatment of DDH often necessitates more invasive treatment, such as open reduction, adductor tenotomy, or osteotomy, to surgically reposition the femoral head [6].

While risk factors for early diagnosed DDH have been well established, risk factors for late diagnosis have not been well described [7-9]. Azzopardi et al. suggest that low birthweight, birth in a rural setting, and hospital discharge less than four days following delivery increase the risk of DDH diagnosis at greater than three months of age [10]. Cold weather and associated increased swaddling in the first few months of life have also been postulated as a risk factor for late DDH diagnosis [11]. By binding the hips in adduction and extension, swaddling may lead to dysplasia in hips that were initially screened as healthy in the newborn infant [12]. Therefore, excessive or improper swaddling in an attempt to keep the infant warm may increase the risk of later dysplasia development, later diagnosis, and subsequent surgical treatment. Lee et al.’s work demonstrated that colder temperatures in the first three months of life increase the risk of surgery for DDH in Taiwan [11]. However, the association between temperature and DDH-related surgery has not been observed in the United States and has not been assessed based on geographic region.

The purpose of this study is to analyze how rates of surgical DDH treatment vary across regions of the United States based on the average outdoor temperatures in January through March, the coldest quarter of the year. 

This study was previously presented as an ePoster at the 2023 IPOS Annual Meeting on December 5-9, 2023.

## Materials and methods

With approval from the Institutional Review Board, a retrospective observational study was conducted using PearlDiver, a large national insurance claims database. New DDH diagnoses from January 2010 to October 2021 were queried using the International Classification of Diseases (ICD)-9 and ICD-10 codes, which are provided in Table 5 in the supplementary material. The following DDH-associated surgical procedures were queried for the same time range with Current Procedural Terminology (CPT) codes: open reduction, adductor tenotomy, femoral osteotomy, and pelvic osteotomy (Table 5). Records for patients five years old or younger with a diagnosis of DDH at the time of treatment were included in the study. Data collection included the patients' sex at birth, state they lived in, and the presence of any risk factors, such as breech birth, low birthweight, oligohydramnios, and Medicaid insurance. Additionally, the type of hip surgery performed was included, such as open reduction, adductor tenotomy, femoral osteotomy, and pelvic osteotomy. The prevalence rates of surgery, defined as the percentage of patients with a DDH diagnosis undergoing surgery out of the total number of DDH patients in the given time range, were calculated per state within the United States. States and associated data were then grouped into five regions with 10 states each, based on average temperatures in the first quarter of the year. These temperatures were reported by the National Center for Environmental Information from 2010 to 2021 [13], and the groups are summarized in Table 1.

**Table 1:** Regional groups according to temperature. Avg: average; Temp: temperature.

Regional group	States included	Avg January-March Temp 2010-2021 (°C)
Group 1 (coldest)	Alaska, North Dakota, Minnesota, Maine, Wisconsin, Vermont, South Dakota, New Hampshire, Michigan, and Montana	-6.17
Group 2 (second coldest)	Wyoming, New York, Iowa, Idaho, Nebraska, Massachusetts, Colorado, Pennsylvania, Connecticut, and Illinois	-1.37
Group 3 (intermediate)	Ohio, Rhode Island, Indiana, Utah, Washington, New Jersey, West Virginia, Oregon, Kansas, and Missouri	1.70
Group 4 (second warmest)	Nevada, Maryland, Delaware, Kentucky, Virginia, New Mexico, Tennessee, Oklahoma, North Carolina, and Arkansas	5.12
Group 5 (warmest)	Arizona, California, South Carolina, Alabama, Mississippi, Georgia, Texas, Louisiana, Florida, and Hawaii	12.37

The rates of surgery in the four colder groups (Groups 1-4) were each compared to that of the warmest group (Group 5) using Pearson’s chi-squared tests. To control for potential confounders (age, biological sex, breech birth, low birthweight, oligohydramnios, and Medicaid payer status), a multivariable logistic regression was conducted to assess the odds of surgery in the four colder groups with respect to the warmest group. Because Medicaid payer status requires an income below the federal poverty line, except for individuals with limited specific conditions, Medicaid payer status was selected as a proxy for socioeconomic status, consistent with prior studies [14,15]. Logistic regression output was recorded using odds ratios and 95% confidence interval (CI). p-values <0.05 were considered statistically significant. Multivariable logistic regression was conducted using R software (R Foundation for Statistical Computing, Vienna, Austria) provided by the PearlDiver database.

## Results

Demographic information

In total, 55,911 patients aged five years or younger with a DDH diagnosis from 2010 to 2021 were identified in the database: 31% male and 69% female. The subgroup of patients who had a surgical intervention for DDH did not differ significantly in gender breakdown. The average age at the time of surgery was 2.30 years. Compared to the entire DDH group, the surgical intervention subgroup had a significantly greater rate of low birthweight (4.72% vs. 2.13%, p<0.001) and oligohydramnios (0.50% vs. 0.26%, p=0.016). In contrast, the surgical subgroup had a lower rate of breech birth (1.94% vs. 5.89%, p<0.001). The rate of Medicaid payer status did not differ between the groups (24.23% vs. 24.40%, p=0.834) (Table 2).

**Table 2:** Characteristics of DDH patients aged 0-5 years. DDH: developmental dysplasia of the hip; SD: standard deviation. ^*^DDH patients were included for analysis at ages 0-5; however, the mean age is not available as the period of observation is over a window of time more than one year.

	DDH patients		Surgical intervention age 0-5 years		p-value
	n	% of total	n	% of total	
Total	55,911	100	2,778	100	
Biological sex					
Male	17,523	31.34	831	29.91	0.114
Female	38,388	68.66	1,947	70.09	
Risk factor					
Breech birth	3,294	5.89	54	1.94	<0.001
Low birthweight	1,189	2.13	131	4.72	<0.001
Oligohydramnios	145	0.26	14	0.50	0.016
Medicaid insurance	13,643	24.40	673	24.23	0.834
			Mean	SD	
Age at surgery^*^			2.30	1.59	

Univariate incidence of surgical intervention before the age of five based on temperature region

**Figure 1:**
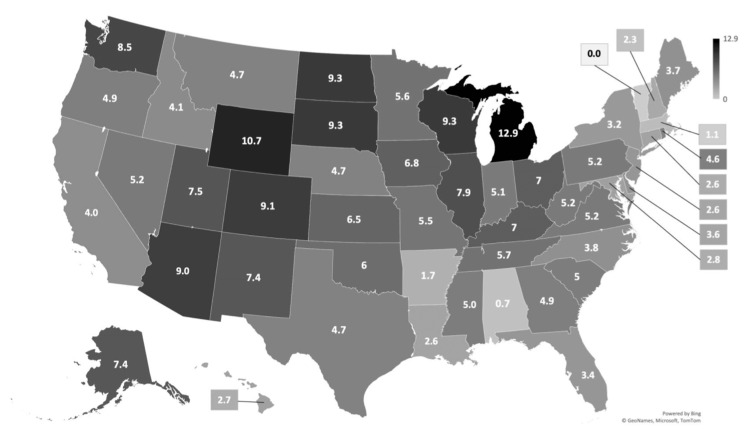
Percentage prevalence of children aged 0-5 who underwent surgical intervention for DDH by state in the United States. DDH: developmental dysplasia of the hip.

The prevalence of surgical intervention for DDH by state is shown in Figure 1. The rates of any surgical intervention were higher for Groups 1 (11.42%) and 3 (5.88%) compared to Group 5 (5.03%; p<0.05 for both). When stratified by surgical intervention type, the rates of open reduction for DDH were higher for Groups 1 (4.59%), 3 (2.50%), and 4 (2.50%) compared to Group 5 (2.06%; p<0.05 for all). The rates of adductor tenotomy, femoral osteotomy, and pelvic osteotomy for DDH were higher for Groups 1 (6.95%, 5.75%, and 5.27%, respectively) and 3 (3.61%, 2.52%, and 2.60%, respectively) compared to Group 5 (2.91%, 2.04%, and 2.04%, respectively; p<0.05 for both) (Table 3).

**Table 3:** Rate of surgical intervention for DDH in children aged 0-5 years according to temperature rank, compared to the 10 warmest states. DDH: developmental dysplasia of the hip. ^*^Pearson's chi-squared test.

Percentage of DDH patients aged 0-5 treated surgically	Group 1 (coldest)	Group 2	Group 3	Group 4	Group 5 (warmest)
Any surgery	11.42%	4.56%	5.88%	5.33%	5.03%
Risk ratio	2.27	0.91	1.17	1.06	1
p-value^*^	<0.001	0.046	0.002	0.37	
Open reduction	4.59%	1.92%	2.50%	2.50%	2.06%
Risk ratio	2.23	0.94	1.21	1.22	1
p-value	<0.001	0.416	0.017	0.036	
Adductor tenotomy	6.95%	2.52%	3.61%	2.94%	2.91%
Risk ratio	2.39	0.87	1.24	1.01	1
p-value	<0.001	0.034	0.001	0.946	
Femoral osteotomy	5.75%	1.85%	2.52%	2.23%	2.04%
Risk ratio	2.82	0.91	1.23	1.1	1
p-value	<0.001	0.247	0.010	0.371	
Pelvic osteotomy	5.27%	1.87%	2.60%	2.29%	2.04%
Risk ratio	2.59	0.92	1.27	1.12	1
p-value	<0.001	0.303	0.003	0.242	

Multivariate likelihood of surgical intervention before the age of five based on temperature region

Multivariable logistic regression analysis demonstrated significantly greater odds of DDH surgery in total and greater odds of each specific procedure in Groups 1-4 compared to Group 5. Patients in Group 1 had over two times the odds of undergoing open reduction or pelvic osteotomy and over three times the odds of undergoing adductor tenotomy or femoral osteotomy compared to Group 5 (Table 4).

**Table 4:** Adjusted odds of surgical interventions for DDH in children aged 0-5 years according to temperature rank, compared to the 10 warmest states. CI: confidence interval; DDH: developmental dysplasia of the hip. ^*^Multivariable logistic regression.

Surgical intervention in DDH patients aged 0-5 years	Odds ratio	95% CI	p-value^*^
First to 10th coldest states			
Any surgery	3.05	[2.92, 3.19]	<0.001
Open reduction	2.50	[2.37, 2.64]	<0.001
Adductor tenotomy	3.44	[3.26, 3.63]	<0.001
Femoral osteotomy	3.37	[3.15, 3.61]	<0.001
Pelvic osteotomy	2.72	[2.56, 2.90]	<0.001
11th-20th coldest states			<0.001
Any surgery	1.31	[1.26, 1.36]	<0.001
Open reduction	1.31	[1.25, 1.38]	<0.001
Adductor tenotomy	1.21	[1.15, 1.27]	<0.001
Femoral osteotomy	1.47	[1.38, 1.56]	<0.001
Pelvic osteotomy	1.21	[1.14, 1.29]	<0.001
21st-30th coldest states			<0.001
Any surgery	1.54	[1.48, 1.60]	<0.001
Open reduction	1.35	[1.28, 1.42]	<0.001
Adductor tenotomy	1.72	[1.65, 1.82]	<0.001
Femoral osteotomy	1.61	[1.52, 1.72]	<0.001
Pelvic osteotomy	1.51	[1.43, 1.61]	<0.001
31st-40th coldest states			<0.001
Any surgery	1.15	[1.10, 1.20]	<0.001
Open reduction	1.15	[1.09, 1.22]	<0.001
Adductor tenotomy	1.24	[1.16, 1.31]	<0.001
Femoral osteotomy	1.26	[1.17, 1.36]	<0.001
Pelvic osteotomy	1.25	[1.17, 1.34]	<0.001

## Discussion

Our results demonstrate that Group 1 patients (coldest) had the highest incidence and likelihood of surgical treatment for DDH before the age of five compared to Group 5 (warmest). These regional differences are likely due to a variety of social and environmental factors, one of which may be outdoor temperature. Lee et al. identified an inverse relationship between the incidence of DDH surgery and the average outdoor temperature [11]. However, contrary to their study, a linear relationship with temperature region and surgical intervention was not noted in this study, as demonstrated by a lower incidence and likelihood of any surgical intervention in Group 2 compared to Group 5. This does not disprove the relationship between temperature and likelihood of surgical treatment for DDH but rather demonstrates that average temperature may only play a role when it is around a threshold of -6.17°C in the coldest quarter of the year, as is the case for the states in Group 1.

The findings of this study also suggest that low birthweight and oligohydramnios were more likely in patients treated surgically than the overall DDH population, although this was not the case for breech birth or Medicaid payer status. Using surgical treatment as a proxy for late DDH diagnosis, this nationwide data suggests that breech birth patients and those on Medicaid are being diagnosed with DDH at an adequate timepoint that allows for non-invasive treatment. This partly aligns with previous studies that have suggested that breech birth, low birthweight, oligohydramnios, and public insurance can all increase the risk of DDH diagnosis [7,10,16,17]. Therefore, improving upon current DDH screening protocols for neonates with low birthweight or oligohydramnios may reduce the need for later surgical intervention.

It has been postulated that the temperature and incidence of surgical management of DDH relationship are related to increased swaddling [11]. By constraining the hips in an adducted and extended position, swaddling can lead to the development of dysplasia in hips that were not dysplastic at birth. Late development of DDH induced by swaddling and subsequent late diagnosis of DDH often necessitate surgical management, as the child is too old to adequately treat non-surgically. The risk of late DDH development can be reduced with proper swaddling technique. Ideally, the infant’s hips are maintained in a natural, flexed position, with freedom of motion throughout the lower extremities at all times [18]. A survey-based study in India reported that 94% of mothers learned swaddling techniques from relatives as opposed to healthcare providers, and their reasons for swaddling included the misbelief that wrapping the legs in an extended position would prevent “bowed legs” [19]. While these exact findings cannot necessarily be translated to the population of parents in the United States, they emphasize the need for primary care providers to accurately and frequently disseminate information about safe swaddling practices with the goal of reducing the incidence of dysplasia development in the first few months of life. It is important to note that our findings cannot be directly linked to increased swaddling in colder regions of the country.

Improvements in screening protocols are necessary for timely DDH diagnosis. Early detection and management of DDH is crucial to avoid long-term sequelae, including early osteoarthritis, pain, and loss of function in the hip. The American Academy of Pediatrics recommends physical exam screening for DDH for all children, from birth through the age of walking [20]. In addition, standard practice in the United States includes ultrasound evaluation for patients at increased risk for dysplasia [20]. Given our study results, the higher likelihood of surgical management for DDH in patients who live in U.S. states with an average first quarter temperature less than -6.17°C may allude to a need for sooner and more frequent ultrasound evaluation and hip surveillance in high-risk patients.

Particularly in U.S. regions with an average first quarter temperature below -6.17°C, there is an increased need to educate new parents and caregivers about the importance of early screening and surveillance for DDH. While some may argue that increased swaddling in cold weather contributes to a higher rate of surgical intervention to manage DDH, the higher incidence of surgical intervention could instead be attributed to parent or caregiver noncompliance and loss of follow-up on DDH diagnosis and management due to severe cold and poor weather conditions. There is limited research regarding how extreme weather affects patient compliance and loss of follow-up specifically in the field of orthopedic surgery. However, Davis et al. reported how weather affected patient visits to the emergency room, showing that cold weather reduced the risk of emergency room visitation by 5-15% at temperatures 10°C or below [21]. They concluded that people may be less willing to seek medical attention on colder days. Other studies have reported similar results with both hot and cold extremes of weather [11,21-23]. Conceptually, there may be a delay in time to initial diagnosis and treatment if parents or caregivers do not regularly follow-up with their pediatrician or orthopedic surgeon due to severe cold temperatures.

While it may appear that a nationwide ultrasound screening and hip surveillance program may be beneficial in the DDH population, from an economic perspective, the effectiveness of universal ultrasound screening in reducing healthcare costs by decreasing surgery rates is debated [24,25]. It is postulated that selective ultrasound screening can be refined to diagnose as many at-risk patients as possible and reduce the need for subsequent surgical procedures while avoiding excessive screening across the entire population [26]. Instead, this study suggests that living in the coldest 10 states in the United States (Alaska, North Dakota, Minnesota, Maine, Wisconsin, Vermont, South Dakota, New Hampshire, Michigan, and Montana) may be a strong enough risk factor to warrant ultrasound screening for DDH, even if no other risk factors are present, which is especially true during the coldest months of the year. Rather than a national program, statewide DDH screening and surveillance programs by primary care physicians and pediatricians during the coldest months of the year may be beneficial.

There are likely additional factors besides temperature that may impact the age at DDH diagnosis and subsequent surgical versus non-surgical treatment. For example, the particularly low surgery rates in Group 2 may suggest the presence of other influential variables. Of note, the Agency for Healthcare Research and Quality reports that all states in Group 2 are at or above average in terms of healthcare quality and access to care [27]. The Chamber of Commerce reports that the three wealthiest states in the country, based on measures such as poverty rate and per capita personal income, are Connecticut, Massachusetts, and New York, all of which are in Group 2 in this study [28]. Therefore, it is possible that high healthcare quality and socioeconomic status enable patients in Group 2 to be diagnosed at a relatively young age on average, despite their region’s cold temperatures.

To our knowledge, this is the first national database study that observes an association between cold weather and DDH treatment in the United States. Nonetheless, our study does have limitations that should be acknowledged before applying to clinical settings. First, the exact age at diagnosis could not be collected, so it was assumed that surgical treatment was an accurate indicator of a late DDH diagnosis. However, it is possible that severe cases of DDH were treated surgically, even if diagnosed early. Second, the utilization of non-operative treatments such as bracing with a Pavlik harness was also not available, and thus, we are unable to determine whether this method was utilized. Third, the causation that cold weather increases the need for surgical intervention cannot be established. Rather, this study reports a correlation between severe cold temperatures and surgical intervention. We postulate that the increased likelihood of surgical intervention during cold temperatures may be related to improper swaddling technique or either irregular or loss of follow-up. However, we are unable to assess whether these truly caused the increased incidence of surgical intervention that was observed. A smaller study involving the collection of swaddling information from parents or a comparison between the birth months of patients with DDH may be beneficial to better support these assertions. Additionally, we used states to differentiate temperature regions. However, there is much temperature variation on a state level that we are unable to differentiate. Lastly, average temperatures may vary by year, with some years being colder than others. While our methods did not include measures of healthcare quality, it would be beneficial to conduct further research observing how these measures vary geographically.

## Conclusions

This study indicates that children living in states with an average temperature near a threshold of -6.17°C in January through March have an increased likelihood of requiring surgical intervention for DDH within the first five years of life. Pediatricians, especially in these colder states, should be aware of potentially higher incidences of DDH and delayed diagnosis, increasing the likelihood of surgical management. Additionally, they should consider the utilization of ultrasound for early diagnosis and regular hip surveillance.

## Appendices

**Table 5:** ICD and CPT codes. ICD: International Classification of Diseases; CPT: Current Procedural Terminology. Source: https://www.aapc.com/codes/

Diagnoses	ICD codes
DDH	ICD-9-D-7543, ICD-9-D-75430, ICD-9-D-75431, ICD-9-D-75432, ICD-9-D-75433, ICD-9-D-75435, ICD-9-D-75463, ICD-10-D-Q65.00, ICD-10-D-Q65.01, ICD-10-D-Q65.02, ICD-10-D-Q65.1, ICD-10-D-Q65.2, ICD-10-D-Q65.30, ICD-10-D-Q65.31, ICD-10-D-Q65.32, ICD-10-D-Q65.4, ICD-10-D-Q65.5, ICD-10-D-Q65.6, ICD-10-D-Q65.9
Breech birth	ICD-9-D-7630, ICD-9-D-6522, ICD-9-D-6696, ICD-10-D-P030
Low birth weight	ICD-9-D-V2130, ICD-9-D-V2131, ICD-9-D-V2132, ICD-9-D-V2133, ICD-9-D-V2134, ICD-9-D-V2135, ICD-10-D-P0700, ICD-10-D-P0701, ICD-10-D-P0702, ICD-10-D-P0703, ICD-10-D-P0710, ICD-10-D-P0714, ICD-10-D-P0715, ICD-10-D-P0716, ICD-10-D-P0717, ICD-10-D-P0718
Oligohydramnios	ICD-9-D-7612, ICD-10-D-P012
Procedures	CPT codes
Open reduction	CPT-27147, CPT-27156, CPT-27258, CPT-27259
Adductor tenotomy	CPT-27000, CPT-27001
Femoral osteotomy	CPT-27140, CPT-27151, CPT-27156, CPT-27161, CPT-27165, CPT-27465, CPT-27466, CPT-27468
Pelvic osteotomy	CPT-27146, CPT-27147, CPT-27151, CPT-27156, CPT-27158
